# Editorial: Women in Colorectal Cancers

**DOI:** 10.3389/fonc.2022.922131

**Published:** 2022-05-10

**Authors:** Francesca De Felice

**Affiliations:** Department of Radiotherapy, Policlinico Umberto I, “Sapienza” University of Rome, Rome, Italy

**Keywords:** colorectal cancer, chemotherapy, screening, treatment, women

The initiative has been launched to promote gender equality. To reach our goals, the first or last author should be a female researcher. It appears that gender equality is still far from being achieved in colorectal cancer publications. Progress in gender equality remains vital. The Research Topics accepted 7 articles including a commentary edited by male authors. The articles can be divided into the following topics:

## Prevention

A contribution deals with preclinical prevention studies in colon cancer. Leystra et al. experimentation focused on molecular processes that are dysregulated in early colon lesions. The hope was to provide valuable baseline data to better design new therapeutic strategies for preventive intervention.

In the clinical setting, the meta-analysis by Xiao et al. indicates that diabetes is associated with a higher risk for right-sided colon cancer than for left-sided colon cancer, suggesting that colonoscopic surveillance in diabetic patients with careful examination of the right colon should be important.

## Biomarkers

Scavo et al. suggested potential novel diagnostic and prognostic biomarker for colorectal cancer and gastric cancer. Bertok et al. described the application of reverse-phase lectin microarrays for the identification of changes in the serum glycome as potential colorectal cancer biomarkers.

In the biomarkers context, it could be useful to consider the Ottaiano and Caraglia opinion. They propose to integrate the diffusion-weighted magnetic resonance in study design to adequately assess the “normalization window” and to perform biomarkers- and tumor volume-based stratification of patients to better interpret results.

## Clinical Practice

Two manuscripts report clinical results of bevacizumab-based therapy. Whether KRAS variants are associated with different clinical behavior is still debated. Giampieri et al. observed that KRAS G12C mutations are associated with worse response rates compared to other KRAS variants when treated with standard chemotherapy doublet plus bevacizumab.

Damato et al. report safety run-in results in the first 10 patients enrolled I the phase II NIVACOR trial. The study was designed to assess the efficacy and safety of nivolumab in combination with chemotherapy triplet scheme (FOLFOXIRI) plus bevacizumab in first-line treatment in patients affected by mCRC RAS/BRAF mutated, regardless of the microsatellite status. The presented preliminary results support the clinicians’ decision to continue the study.

## Author Contributions

The author confirms being the sole contributor of this work and has approved it for publication.

## Conflict of Interest

The author declares that the research was conducted in the absence of any commercial or financial relationships that could be construed as a potential conflict of interest.

## Publisher’s Note

All claims expressed in this article are solely those of the authors and do not necessarily represent those of their affiliated organizations, or those of the publisher, the editors and the reviewers. Any product that may be evaluated in this article, or claim that may be made by its manufacturer, is not guaranteed or endorsed by the publisher.

